# Learnability of a Configurator Empowering End Users to Create Mobile Data Collection Instruments: Usability Study

**DOI:** 10.2196/mhealth.9826

**Published:** 2018-06-29

**Authors:** Johannes Schobel, Rüdiger Pryss, Thomas Probst, Winfried Schlee, Marc Schickler, Manfred Reichert

**Affiliations:** ^1^ Institute of Databases and Information Systems Ulm University Ulm Germany; ^2^ Department for Psychotherapy and Biopsychosocial Health Danube University Krems Krems Austria; ^3^ Department of Psychiatry and Psychotherapy University of Regensburg Regensburg Germany

**Keywords:** mHealth, data collection, mobile apps

## Abstract

**Background:**

Many research domains still heavily rely on paper-based data collection procedures, despite numerous associated drawbacks. The QuestionSys framework is intended to empower researchers as well as clinicians without programming skills to develop their own smart mobile apps in order to collect data for their specific scenarios.

**Objective:**

In order to validate the feasibility of this model-driven, end-user programming approach, we conducted a study with 80 participants.

**Methods:**

Across 2 sessions (7 days between Session 1 and Session 2), participants had to model 10 data collection instruments (5 at each session) with the developed configurator component of the framework. In this context, performance measures like the time and operations needed as well as the resulting errors were evaluated. Participants were separated into two groups (ie, novices vs experts) based on prior knowledge in process modeling, which is one fundamental pillar of the QuestionSys framework.

**Results:**

Statistical analysis (*t* tests) revealed that novices showed significant learning effects for errors (*P*=.04), operations (*P*<.001), and time (*P*<.001) from the first to the last use of the configurator. Experts showed significant learning effects for operations (*P*=.001) and time (*P*<.001), but not for errors as the experts’ errors were already very low at the first modeling of the data collection instrument. Moreover, regarding the time and operations needed, novices got significantly better at the third modeling task than experts were at the first one (*t* tests; *P*<.001 for time and *P*=.002 for operations). Regarding errors, novices did not get significantly better at working with any of the 10 data collection instruments than experts were at the first modeling task, but novices’ error rates for all 5 data collection instruments at Session 2 were not significantly different anymore from those of experts at the first modeling task. After 7 days of not using the configurator (from Session 1 to Session 2), the experts’ learning effect at the end of Session 1 remained stable at the beginning of Session 2, but the novices’ learning effect at the end of Session 1 showed a significant decay at the beginning of Session 2 regarding time and operations (*t* tests; *P*<.001 for time and *P*=.03 for operations).

**Conclusions:**

In conclusion, novices were able to use the configurator properly and showed fast (but unstable) learning effects, resulting in their performances becoming as good as those of experts (which were already good) after having little experience with the configurator. Following this, researchers and clinicians can use the QuestionSys configurator to develop data collection apps for smart mobile devices on their own.

## Introduction

In psychology and social sciences, *self-report questionnaires* are commonly used to collect data in various situations [1]. These data are predominantly collected using *paper-based* questionnaires, which are costly regarding the subsequent processing and analysis of the collected data. Furthermore, the latter has to be transferred to digital spreadsheet documents, which is a time-consuming and error-prone task, especially in the context of large-scale trials or studies. According to one estimate, approximately 50%-60% of the costs related to the collection, transfer, and processing of the data could be saved using digital instruments instead of paper-based ones [2]. Additionally, electronic questionnaires do not differ from the paper-based versions in psychometric properties [3]. Moreover, they contribute to more complete datasets compared with the ones collected using pencil and paper [4], resulting in a better data quality [5]. Finally, the digitally collected data may be enriched with contextual information [6] (eg, time and location) or sensor data [7] (eg, pulse measurement during an interview). In general, digital instruments are in increasing demand to support clinical trials or other psychological studies [8].

Over the last decade, several Web-based questionnaire apps (eg, *LimeSurvey* or *SurveyMonkey*) emerged, enabling end users to create online questionnaires themselves. Although these apps are useful, they are not suitable in certain application scenarios. Among others, Web questionnaires require permanent internet access and are usually unable to capture data from external sensors (eg, camera, Global Positioning System, or vital parameter sensors). *Smart mobile devices* (eg, mobile phones or tablets), in turn, could act as an enabler for scenarios in which Web questionnaires are not sufficient. Mobile devices have already proven their applicability in the context of various business scenarios [9], ranging from simple task management apps to sophisticated business analytics platforms to even apps supporting ward rounds in hospitals [10].

Contrary to these findings, however, mobile data collection apps are still rarely used in large-scale scenarios, like clinical or psychological trials. The following three reasons are of paramount importance in this context:

1. Researchers are unaware of the capabilities of and opportunities offered by smart mobile devices in their respective domain. This can also be traced back to the high costs of such devices, especially in the context of large-scale studies requiring multiple devices.
2. Already existing data collection apps do not adequately support researchers. There might be legal aspects that need to be considered (eg, “Where shall the data be stored?”, “Who shall be allowed to access the data?”); the mobile apps might require permanent internet access, or their advanced features (eg, use of sensors during the data collection procedure) need to be supported.
3. Implementing sophisticated mobile data collection apps usually requires considerable communication efforts between researchers and mobile app developers. This communication is further aggravated due to the fact that both groups use different *languages* (ie, terminology, or [graphical] notations) to express themselves.

It is noteworthy that there are several mobile apps proving the applicability of smart mobile devices in the context of data collection scenarios, such as *Manage My Pain* [11] or *Track Your Tinnitus* [12]. Although the participants involved in respective scenarios gave positive feedback, several shortcomings could still be observed. The latter include, for example, high development costs, the need for skilled app developers, or the common business-IT alignment gap (ie, domain experts being unable to express what developers shall realize) [13]. When relying purely on smart mobile devices for data collection purposes, specific participant groups may be excluded (eg, elderly) [14]. Furthermore, providing respective mobile app for only one mobile operating system (eg, Android or iOS) might result in biased samples as their users can differ regarding various aspects such as income, age, or education [15].

We also observed these issues in several long-running, large-scale data collection scenarios for which we had provided mobile apps (see Table 1).

**Table 1:** Implemented mobile data collection apps.

Data collection scenario	Country	Complex navigation^a^	Duration (years)	App versions	Collected datasets using smart mobile apps
Study on tinnitus research [17]	Worldwide	No	>5	5	≥45,000
Risk factors during pregnancy [18]	Germany	No	>5	5	≥1500
Risk factors after pregnancy	Germany	No	>2	1	≥500
Posttraumatic stress disorder in war regions [19]	Burundi	Yes	4	5	≥2200
Posttraumatic stress disorder in war regions [20]	Uganda	No	1	1	≥200
Adverse childhood experiences [21]	Germany	Yes	2	3	≥150
Learning deficits among medical students	Germany	Yes	1	3	≥200
Supporting parents after accidents of children	European Union	No	>3	6	≥5000
Overall	—	—	—	29	≥54,750

^a^No: complex navigation was not requested/required; yes: complex navigation was requested/required.

**Figure 1:**
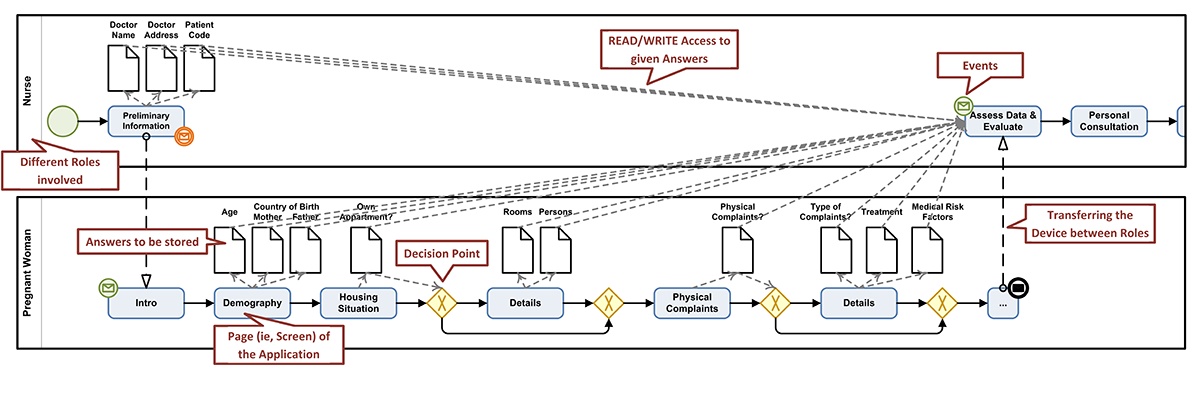
A data collection instrument represented as BPMN 2.0 model.

Most of these apps were explicitly tailored and implemented to support a specific application scenario. Developing such a plethora of data collection instruments enabled us to elaborate crucial requirements in this context [16]. Although the involved investigators and clinicians were satisfied with the provided mobile data collection apps, they craved for more sophisticated features over time. The latter include, for example, audio-recordings during interviews, additional notes, and real-time data analyses. Furthermore, maintaining these specifically implemented apps over time was a costly and time-consuming endeavor. In order to relieve app developers from such tasks, researchers as well as clinicians should be enabled to develop mobile apps themselves. Existing approaches [22,23] combine *WordPress*, a blogging software, and *iBuildApp*, a Web-based app builder, to create a platform supporting students from clinical psychiatry. The focus of this platform, however, is on information retrieval (eg, psychiatric guidelines). Furthermore, only limited support regarding the development of digital instruments is provided. Other projects like *MagPi* or *MovisensXS* also provide configurators using simple Web forms, allowing end users to create data collection apps. Our work significantly differs from these approaches as we focus on sophisticated data collection instruments based on advanced *process management technology*. In particular, well-established graphical notations are provided to express various aspects of such data collection instruments. Figure 1 represents an instrument using the Business Process Modeling and Notation (BPMN) 2.0 graphical notation [24] that provides a solid basis for the developed configurator. The latter, however, uses its own graphical notation in order to allow end users without expertise in process modeling to apply such techniques. The modeled instrument, in turn, may then be executed on smart mobile devices, such as mobile phones or tablets.

In general, graphical process notations comprise various elements that allow specifying and visualizing complex business processes in enterprises (eg, partners involved and their roles in the process, data elements, or temporal process constraints). When applying such a notation to the modeling of data collection instruments, several issues emerged. In particular, researchers were overwhelmed by the multitude of graphical elements as well as their semantical meaning needed to properly represent their specific data collection instrument. More precisely, dealing with data elements was especially challenging when modeling such instruments. First, the data element needs to be specified accordingly. Second, a question that produces (ie, writes) this data element must be modeled; third, the data element must be consumed (ie, read) by decisions later in order to properly control the flow of the instrument. Monitoring these aspects, while still dealing with the modeling process in general, is challenging.

To make such an expressive *modeling approach* better accessible for end users with little or no knowledge of process modeling (ie, researchers or clinicians), end-user programming techniques were evaluated. Such techniques, in turn, have proven their feasibility in a multitude of studies to support nonprogrammers. The use of a graphical programming language instead of a text-based one has been evaluated to teach children programming [25]. Their teachers reported that the simplified representation significantly improved the understanding of program code. Another approach [26] applied end-user programming techniques to support administrators in “writing” management scripts used in their daily routines. The “programming” of Web Mashups, which combines operators and functions in a graphical manner, has been previously presented [27]. Among others, these studies have proven the applicability of end-user programming approaches in their specific domain.

Taking the above issues into account, the *QuestionSys* configurator that we developed applies sophisticated end-user programming techniques to properly abstract the modeling of data collection instruments. Accordingly, QuestionSys offers a user-friendly configurator, hiding most of the complexity introduced by process modeling languages. Particularly, this configurator uses its own (graphical) modeling notation based on BPMN 2.0 in order to allow end users without any programming skills or knowledge in process modeling to graphically specify data collection instruments themselves. Therefore, there should be no need for involving IT experts anymore when developing such mobile data collection instruments [28]. To be more precise, QuestionSys particularly focuses on scenarios in which the instruments need to be frequently adapted. Especially these adaptations shall be accomplished by end users with no programming experience in order to reduce costs. 

Frequent adaptations, in turn, require the continuous use of the respective configurator. Especially usability of apps that are used in a daily manner is of paramount importance. One aspect that is relevant in this context is the prior experience needed to learn the app. Following this, it is important for the usability of an app to assess learnability.

For this purpose, in a pilot study, we evaluated the QuestionSys configurator with 44 participants and obtained promising results with respect to the modeling of data collection instruments and overall usability of the configurator [29]. We found that individuals with no experience in process modeling understood how to properly use the configurator. Based on this pilot study, we conducted a larger study with a more sophisticated study design that comprised 2 testing sessions (second session 7 days after the first one) with 5 tasks (ie, modeling data collection instruments) at each session. This refined study and its results are presented in the manuscript at hand. The following research questions (RQs) were addressed with *novices* (ie, individuals with no experience in process modeling) and *experts* (individuals with experience in process modeling):

RQ1: How are the performances of novices and experts changing from the first to the last task (data collection instrument) of Session 1?

RQ2: How are the performances of novices and experts changing from the last task (data collection instrument) of Session 1 to the first task (data collection instrument) of Session 2?

RQ3: How are the performances of novices and experts changing from the first to the last task (data collection instrument) of Session 2?

RQ4: How are the performances of novices and experts changing from the first task (data collection instrument) of Session 1 to the last task (data collection instrument) of Session 2?

RQ5: How many tasks (data collection instruments) are necessary until the performance metrics of novices are as good as those of experts at the first task (data collection instrument)?

## Methods

### Configurator Component

**Figure 2:**
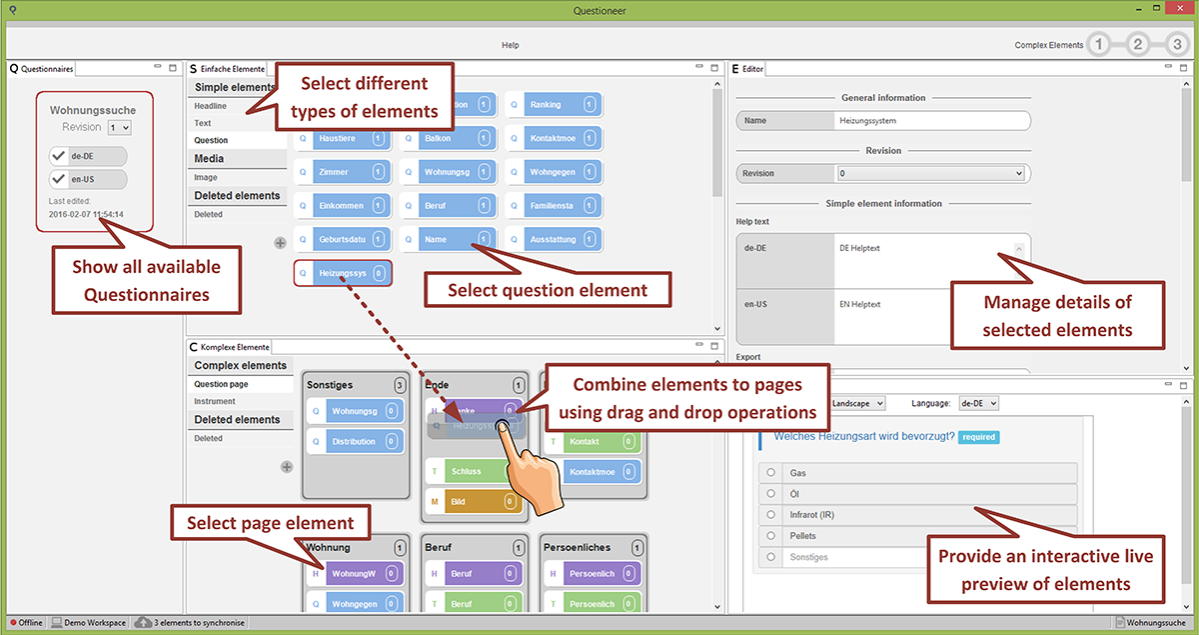
The QuestionSys configurator: combining elements to pages.

**Figure 3:**
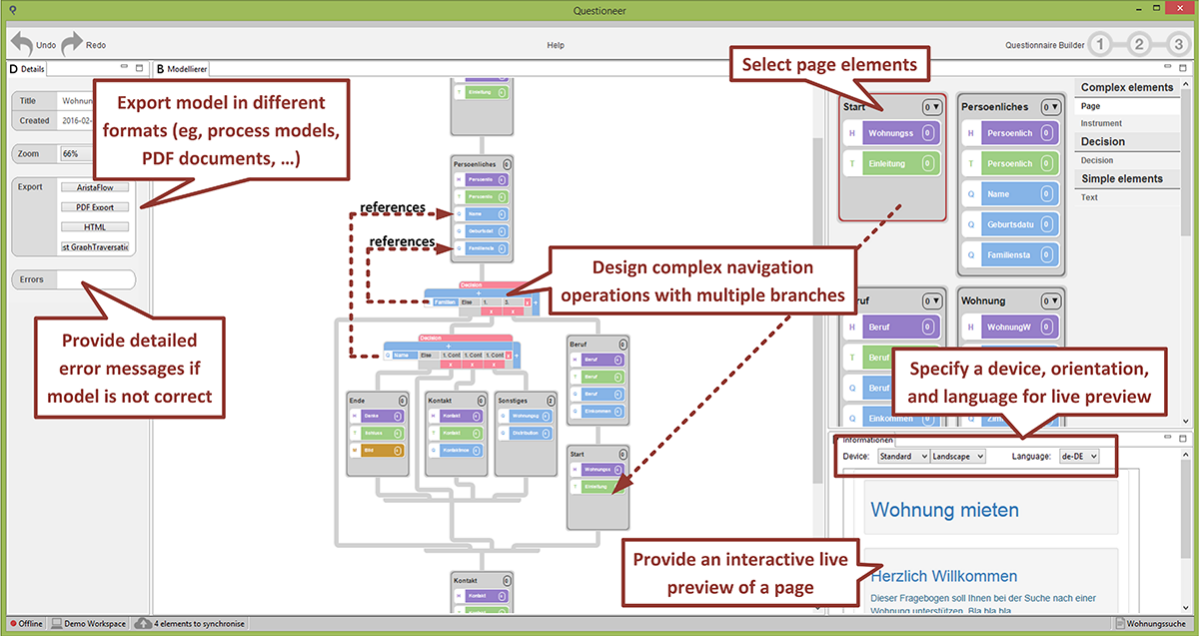
The QuestionSys configurator: modeling a data collection instrument.

The combined use of well-known technologies from end-user programming and business process management enables end users to create mobile data collection instruments on their own. The most important views of the configurator component (“Element and Page Repository View” and “Modeling Area View”) are sketched in Figures 2 and 3; see [30] for more details.

Element and Page Repository View (see Figure 2). The element repository allows creating basic elements of a questionnaire (eg, texts and questions). The rightmost part shows an editor panel, where particular attributes of the respective elements may be managed. The configurator enables researchers to handle elements in multiple languages and revisions. Most importantly, the created elements may be combined to pages using drag & drop operations.

Modeling Area View (see Figure 3). Previously created pages may be used to model the structure of the data collection instrument. Furthermore, researchers are able to model advanced navigation operations (eg, to *skip* pages depending on already given answers to previous questions) to adapt the instrument during the data collection process. The modeling view, in turn, provides guidance for untrained users; particularly, it does not allow applying *wrong* operations to the model. Note that the QuestionSys configurator applies its own (graphical) modeling notation. The latter, however, is inspired by BPMN 2.0, but significantly simplifies the modeling process for individuals having no experience with process modeling notations (eg, no explicit data flow needs to be modeled).

Altogether, the configurator component and its model-driven approach allow researchers to graphically define the elements and logic of data collection instruments.

In order to be able to automatically collect the data needed for the evaluation of the configurator component, the latter was enhanced with a *Study Mode* that enables specific features. First, it requires users to enter a *code* before using the configurator. This code, in turn, is used to store all collected data in a dedicated folder. Second, the configurator tracks the *time* when a specific *operation* (eg, adding a page to the model) was performed. Third, after performing the operation, an image of the model is stored on the computer. This allows reproducing the *process of modeling* a data collection instrument step-by-step as well as manually evaluating the *errors* in the resulting model.

### Study Procedure

Participants modeled a series of data collection instruments (ie, 5 data collection instruments per session) with the QuestionSys configurator over 2 sessions (with 7 days between Session 1 and Session 2). A controlled environment was chosen for this study in order to be able to quickly react to upcoming problems. For the study, 20 workstations, each comparable in hardware resources (eg, RAM and central processing unit cores), were prepared in a computer pool at Ulm University. Each workstation was equipped with two monitors running a common resolution. Before each of the 2 sessions, respective workstations were prepared carefully. This includes, for example, reinstalling the configurator component and placing the consent form, description of tasks, and mental effort questionnaires next to the workstation.

**Figure 4:**
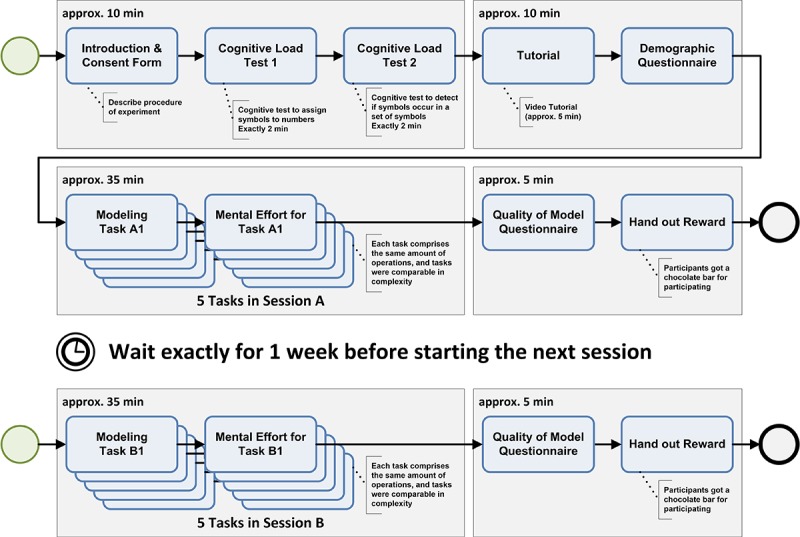
Study design.

The procedure of the study is outlined in Figure 4. The study started with welcoming the participants and introducing the goal of the study as well as the overall procedure. Then, the participants performed 2 tests (2 min each) measuring their processing speed. Both tests are reliable and valid tests of the *Wechsler Adult Intelligence Scale* [31]. Next, we provided a tutorial (approximately 5 min) demonstrating the most important features of the configurator. Before conducting the main part of the study, the participants were asked to fill in a demographic questionnaire. Up to this point in time, the participants were allowed to ask questions. Next, participants had to model 5 data collection instruments (tasks; see Table 2) using solely the provided configurator component, followed by filling in a short questionnaire. Concluding the first session, participants had to answer a short questionnaire again. Altogether, this first session took approximately 50 min.

After pausing for exactly 1 week, the participants were reinvited for a second session. The latter, however, was much shorter as the collection of demographic data could be skipped. Participants were given 5 new tasks (ie, to model 5 new data collection instruments; see Table 2), and they had to answer the short questionnaires again.

The data, automatically recorded by the configurator, were then uploaded to a network-attached storage after each session.

**Table 2:** Short description of the tasks to be modeled by participants.

#	Modeling a questionnaire	Pages	Decisions
1	...to collect information about flight passengers	5	2
2	...to help customers select an appropriate mobile phone	5	2
3	...to help collect required information for travel expense reports	5	2
4	...to order food and drinks online	5	2
5	...to support customers select a movie and book cinema tickets	5	2
6	...to help customers select an appropriate laptop computer	5	2
7	...to support customers book seats for a theater play	5	2
8	...to inform patients regarding their upcoming surgery	5	2
9	...to guide customers through the process of purchasing a new coffee machine and equipment	5	2
10	...to collect required data to conclude a contract in a gym	5	2

All materials and methods were approved by the Ethics Committee of Ulm University and were carried out in accordance with the approved guidelines. All participants gave their informed consent.

### Tutorial

Before working with the configurator app, a screencast tutorial was presented directly to each participant. The latter was recorded by us; it describes how to create a very simple data collection instrument. No voice or sound was recorded; however, the screencast was annotated with small comments in postproduction.

### Tasks

Each task to be modeled was presented in a textual representation that described the overall structure of the data collection instrument to be created.

When designing the tasks for the participants, we paid close attention to the fact that all 10 tasks were mutually comparable. As this study intended to measure the learnability, which is a contributory factor of the overall usability of the configurator, it was of utmost importance to keep the complexity for all modeling tasks constant. Tasks in divergent complexity, in turn, may limit the validity of the study results as a change in performance measures may be attributed to a more complex model itself or respective learning effect. The overall complexity includes, on one hand, the complexity of the textual representation handed out to the participants and, on the other, the complexity of the resulting data collection instrument. 

All tasks were designed so that a perfect instrument modeling solution would need exactly the same number of operations. Furthermore, each model contained two decision points in order to influence the further processing of the instrument based on given answers. Thematically, the models to be created were selected from various domains, ranging from a health care instrument up to a questionnaire for a food delivery service (see Table 2).

### Participants

In total, 80 participants were recruited at Ulm University for the experiment. Most of them were students or research associates from various departments, like computer science, economics, chemistry, psychology, and medicine [32]. We recruited participants from these different disciplines to allow a comparison of how individuals with no experience in process modeling can learn using the configurator compared with individuals with experience in process modeling. The target group (end users from medical, psychological, or social sciences) that shall be empowered by the configurator to develop mobile data collection instruments has most probably no experience in process modeling. During the recruitment phase, we paid close attention to maintaining the balance between female and male participants. Students willing to participate were instructed according to the developed study design, which was explained to them before, and they were additionally informed that there will be 2 consecutive sessions to attend. To ensure that all participants correctly understood the tasks to be performed by them, all relevant material was handed out in German [33]. Participants who answered the prequestion (ie, a question in the demographic questionnaire) *“Do you have experience in process modeling”* with *yes* were classified as *experts*, whereas participants who answered this question with *no* were classified as *novices*. It should be kept in mind that this is only one (simplified) possibility to classify participants into *novices* and *experts*. Another possibility would be more in-depth prequestioning of participants (eg, asking about familiarity with notations such as BPMN, asking for examples of process models they have created, and asking specific questions about particular items in process modeling notations). This would lead to a spectrum of rated expertise, rather than the simplified binary approach used in this study.

Altogether, our classification resulted in 45 novices and 35 experts. Three of the recruited participants did not participate in the second session (1 novice and 2 experts). Therefore, RQs 2-4 (RQs that included data gathered in the second session) were investigated with 77 participants (44 novices and 33 experts).

### Baseline Measures

To evaluate whether experts and novices differed in their cognitive abilities, we performed 2 established tests measuring their processing speed [31]. Within 2 min each, participants had to assign symbols to numbers (“Digital Symbol Coding”) and detect symbols from within a set of symbols (“Symbol Search”). Differences in cognitive abilities at baseline would be a confounder as a higher cognitive ability could result in better or faster learnability of the configurator.

### Questionnaires

A demographic questionnaire collecting personal information (eg, gender or education) was handed out to the participants. Specific focus was put on questions about their prior knowledge regarding process modeling, in general, or about how many process models they had read and written during the last 12 months, in particular. After completing each task, participants had to answer 5 questions regarding their mental effort when modeling the instrument. At the end of each session, they had to answer questions regarding the quality of the modeled instruments or their own competence when working with the provided configurator component.

### Performance Measures

The following performance measures were collected:

#### Time

The moment participants started modeling their instruments, the respective timestamp was added to an Excel file stored in the configurator app’s directory. Once the task was completed, another timestamp was added to the file. This allowed us to evaluate the time taken to complete the respective tasks on a fine-grained level (values were assessed in milliseconds).

#### Operations

Whenever participants interacted with the instrument (eg, by adding a new page), their specific operations were logged in an Excel file. In addition, the time at which the participant executed this operation was logged. Finally, the configurator took an image of the current model after performing the respective operation and stored it to the directory of the participant.

#### Errors

It was not possible for the configurator to automatically assess the errors in the resulting model (eg, order of branches in decision points may be switched or respective statements may be inverted). Therefore, we manually evaluated all created models based on the provided images. As the configurator provided a snapshot of the model after each operation, it was possible to recreate the modeling process of each participant. Furthermore, this allowed us to assess the models on a fine-grained basis.

### Statistics

SPSS 23 was used for all statistical analyses. Frequencies, percentages, means, and standard deviations were calculated as descriptive statistics. Novices and experts were compared in baseline variables using Fisher’s exact tests and *t* tests for independent samples. To test RQs 1-4, *t* tests for dependent samples were performed to investigate the change in the performance measures between the tasks (data collection instruments) specified in the corresponding RQ; these *t* tests for dependent samples, in turn, were conducted separately for novices and experts. *t* tests for independent samples were performed to evaluate RQ5, thereby performances of novices at each task (data collection instrument) were compared with those of experts at Task 1 of Session 1 (first data collection instrument) in order to identify the tasks (data collection instruments) for which the performances of novices were not significantly different from those of experts at Task 1 of Session 1 (first data collection instrument). All statistical tests were performed two tailed; the significance value was set to *P*<.05.

### Data Availability

The raw data set containing all collected data that was analyzed during this study is included in this paper (and its supplementary material).

## Results

### Baseline Comparison Between Novices and Experts

Table 3 summarizes the sample description and comparisons between novices and experts in baseline variables. There were more female participants in the novices’ sample and more male participants in the experts’ sample (*P*=.003). Moreover, the experts’ sample comprised a higher percentage of participants with a bachelor degree as highest education than the novices’ sample, whereas the latter comprised a higher percentage of participants with graduating high school as highest education (*P*=.009). While a higher percentage of the novices’ sample studied psychology than the experts’ sample, a higher percentage in the latter studied economics or computer science than in the former (*P*=.001).

**Table 3:** Sample description and comparison between novices and experts in baseline variables.

Variable		Novices (n=45)	Experts (n=35)		*P* value
**Gender, n (%)**					.003^a^
	Female	31 (69)		12 (34)	
	Male	14 (31)		23 (66)	
Age (years), mean (SD)		21.20 (2.63)	22.72 (2.97)		.180^a^
**Age category, n (%)**					
	<25 years	29 (64)		17 (49)	
	25-35 years	16 (36)		18 (51)	
**Highest education, n (%)**					.009^a^
	High school	13 (29)		2 (6)	
	Bachelor	32 (71)		32 (91)	
	Master	0 (0)		1 (3)	
**Current field of study, n (%)^b^**					.001^a^
	Economics	14 (33)		12 (40)	
	Media computer science	0 (0)		8 (27)	
	Computer science	1 (2)		6 (20)	
	International business	0 (0)		1 (3)	
	Chemistry	2 (5)		0 (0)	
	Psychology	26 (60.5)		3 (10)	
**Processing speed test 1: digital symbol coding, mean (SD)**					
	Correct answers	84.33 (21.76)		81.11 (21.89)	.515
	Wrong answers	0.07 (0.25)		0.06 (0.24)	.864
**Processing speed test 2: symbol search, mean (SD)**					
	Correct answers	41.93 (7.77)		38.91 (8.53)	.103
	Wrong answers	1.73 (1.98)		1.63 (1.50)	.795

^a^Fisher’s exact test.

^b^N=73/80 (91%) participants gave information on their current field of study.

### Results for RQ1

#### Time

Novices (n=45): The mean time (in milliseconds) required for the first task of Session 1 (first data collection instrument) was 452,334.29 (SD 209,527.70), and the mean time required for the last task of Session 1 (fifth data collection instrument) was 135,273.89 (SD 49,861.64). This improvement reached statistical significance: *t* (44)=10.71; *P*<.001.

Experts (n=35): The mean time needed (in milliseconds) for a task significantly decreased from 405,444.89 (SD 248,497.68) at the first task of Session 1 (first data collection instrument) to 147,251.91 (SD 91,181.39) at the last task of Session 1 (fifth data collection instrument): *t* (34)=6.12; *P*<.001.

#### Operations

Novices (n=45): Operations significantly decreased from a mean 17.60 (SD 7.87) at the first task of Session 1 (first data collection instrument) to 11.24 (SD 3.68) at the last task of Session 1 (fifth data collection instrument): *t* (44)=5.23; *P*<.001.

Experts (n=35): Significantly less operations were needed at the last task of Session 1 (fifth data collection instrument) than at the first task of Session 1 (first data collection instrument): 17.49 (SD 11.20) at the first task of Session 1 and 11.31 (SD 3.98) at the last task of Session 1: *t* (34)=3.41; *P*=.002.

#### Errors

Novices (n=45): Errors nonsignificantly decreased from 1.24 (SD 2.15) at the first task of Session 1 (first data collection instrument) to 1.00 (SD 1.83) at the last task of Session 1 (fifth data collection instrument): *t* (44)=0.88; *P*=.386.

Experts (n=34, as errors were not available for one expert because of corrupted snapshot images): Errors decreased from a mean of 0.35 (SD 0.88) at the first task of Session 1 (first data collection instrument) to 0.32 (SD 0.84) at the last task of Session 1 (fifth data collection instrument). However, this change was not statistically significant: *t* (33)=0.16; *P*=.876.

### Results for RQ2

#### Time

Novices (n=44): The mean time (in milliseconds) significantly increased from the last task of Session 1 (fifth data collection instrument) to the first task of Session 2 (sixth data collection instrument): 133,725.80 (SD 49,332.01) versus 235,291.93 (SD 167,630.02); *t* (43)=−3.82; *P*<.001.

Experts (n=33): No significant change in the mean time (in milliseconds) emerged between the last task of Session 1 (fifth data collection instrument) and the first task of Session 2 (sixth data collection instrument): 148,253.30 (SD 93,726.57) versus 222,304.67 (SD 227,425.64); *t* (32)=−1.76; *P*=.088.

#### Operations

Novices (n=44): Significantly more operations were observed at the first task of Session 2 (sixth data collection instrument) than at the last task of Session 1 (fifth data collection instrument): 11.11 (SD 3.61) versus 13.89 (SD 6.88); *t* (43)=−2.25; *P*=.030.

Experts (n=33): Operations did not significantly change between the last task of Session 1 (fifth data collection instrument) and the first task of Session 2 (sixth data collection instrument): 10.97 (SD 3.62) versus 12.70 (SD 5.93); *t* (32)=−1.46, *P*=.155.

#### Errors

Novices (n=44): Errors did not significantly change between the last task of Session 1 (fifth data collection instrument) and the first task of Session 2 (sixth data collection instrument): 1.02 (SD 1.85) versus 0.86 (SD 1.44); *t* (43)=0.69; *P*=.492.

Experts (n=33): From the last task of Session 1 (fifth data collection instrument) to the first task of Session 2 (sixth data collection instrument), errors did not significantly change: 0.33 (SD 0.85) versus 0.46 (SD 0.97); *t* (32)=−0.61; *P*=.545.

### Results for RQ3

#### Time

Novices (n=44): The mean time (in milliseconds) significantly decreased from the first task of Session 2 (sixth data collection instrument), 235,291.93 (SD 167,630.02), to the last task of Session 2 (tenth data collection instrument), 107,957.18 (SD 54,837.64): *t* (43)=5.12; *P*<.001.

Experts (n=33): The mean time (in milliseconds) significantly decreased from the first task of Session 2 (sixth data collection instrument), 222,304.67 (SD 227,425.64), to the last task of Session 2 (tenth data collection instrument), 85,600.36 (SD 23,698.01): *t* (32)=3.53; *P*=.001.

#### Operations

Novices (n=44): Operations became significantly less from the first task of Session 2 (sixth data collection instrument), 13.89 (SD 6.88), to the last task of Session 2 (tenth data collection instrument), 11.55 (SD 4.86): *t* (43)=2.01; *P*=.050.

Experts (n=33): Significantly less operations were needed at the last task of Session 2 (tenth data collection instrument), 9.45 (SD 1.06), than at the first task of Session 2 (sixth data collection instrument), 12.70 (SD 5.93): *t* (32)=3.00; *P*=.005.

#### Errors

Novices (n=44): Errors did not significantly change between the first task (sixth data collection instrument), 0.86 (SD 1.44), and last task (tenth data collection instrument), 0.64 (SD 1.01), of Session 2: *t* (43)=1.26; *P*=.215.

Experts (n=33): No change in errors between the first task (sixth data collection instrument), 0.46 (SD 0.97), and last task (tenth data collection instrument), 0.30 (SD 0.85), of Session 2 emerged: *t* (32)=0.78; *P*=.443.

### Results for RQ4

#### Time

Novices (n=44): From the first task of Session 1 (first data collection instrument) to the last task of Session 2 (tenth data collection instrument), the mean time (in milliseconds) significantly decreased: 456,322.02 (SD 210,215.59) versus 107,957.18 (SD 54,837.64); *t* (43)=11.30; *P*<.001.

Experts (n=33): The mean time (in milliseconds) significantly decreased from the first task of Session 1 (first data collection instrument) to the last task of Session 2 (tenth data collection instrument) 393,204.06 (SD 46,642.43) versus 85,600.36 (SD 23,698.01); *t* (32)=7.24; *P*<.001.

#### Operations

Novices (n=44): From the first task of Session 1 (first data collection instrument) to the last task of Session 2 (tenth data collection instrument), operations became significantly less: 17.80 (SD 7.85) versus 11.55 (SD 4.86); *t* (43)=4.98; *P*<.001.

Experts (n=33): Operations significantly decreased from the first task of Session 1 (first data collection instrument) to the last task of Session 2 (tenth data collection instrument): 17.18 (SD 11.41) versus 9.45 (SD 1.06); *t* (32)=3.83; *P*=.001.

#### Errors

Novices (n=44): Errors significantly decreased from the first task of Session 1 (first data collection instrument) to the last task of Session 2 (tenth data collection instrument): 1.7 (SD 2.17) versus 0.64 (SD 1.01); *t* (43)=2.09; *P*=.043.

Experts (n=33): Errors did not significantly change between the first task of Session 1 (first data collection instrument) and the last task of Session 2 (tenth data collection instrument): 0.36 (SD 0.90) versus 0.30 (SD 0.85); *t* (32)=0.30; *P*=.768.

### Results for RQ5

#### Time

The comparisons between the time (in milliseconds) of experts at the first task of Session 1 and the time of novices at each task are presented in Table 4. It can be seen that novices were not significantly different from experts already at the first task of Session 1 (first data collection instrument) and that the time taken by novices at Tasks 3-10 was significantly less than that taken by experts at Task 1 of Session 1 (*P*=.363 comparing Task 1 of novices with Task 1 of experts; *P*=.062 comparing Task 2 of novices with Task 1 of experts; *P*<.001 comparing Task 3 of novices with Task 1 of experts; *P*<.001 comparing Task 4 of novices with Task 1 of experts; *P*<.001 comparing Task 5 of novices with Task 1 of experts; *P*=.001 comparing Task 6 of novices with Task 1 of experts; *P*<.001 comparing Task 7 of novices with Task 1 of experts; *P*<.001 comparing Task 8 of novices with Task 1 of experts; *P*<.001 comparing Task 9 of novices with Task 1 of experts; *P*<.001 comparing Task 10 of novices with Task 1 of experts).

#### Operations

Table 5 compares the operations of experts at the first task of Session 1 and those of novices at each task. Again, novices performed not significantly different from experts already at the first task of Session 1 (first data collection instrument). Moreover, the operations of novices at Tasks 3, 4, 5, 7, 8, 9, and 10 were significantly less than those of experts at Task 1 of Session 1. Only the difference between operations of novices at Task 6 (first data collection instrument of Session 2) and those of experts at Task 1 (first data collection instrument of Session 1) did not reach statistical significance (*P*=.957 comparing Task 1 of novices with Task 1 of experts; *P*=.373 comparing Task 2 of novices with Task 1 of experts; *P*=.002 comparing Task 3 of novices with Task 1 of experts; *P*=.027 comparing Task 4 of novices with Task 1 of experts; *P*=.003 comparing Task 5 of novices with Task 1 of experts; *P*=.101 comparing Task 6 of novices with Task 1 of experts; *P*=.007 comparing Task 7 of novices with Task 1 of experts; *P*=.004 comparing Task 8 of novices with Task 1 of experts; *P*=.020 comparing Task 9 of novices with Task 1 of experts; *P*=.005 comparing Task 10 of novices with Task 1 of experts).

**Table 4:** Comparisons between the time (in milliseconds) taken by experts at the first task of Session 1 and that taken by novices at each task.

Sample and task		Session	N	Mean (SD)	*P* value^a^
**Experts**					
	Task 1	1	35	405,444*.* 89 (248,497*.* 68)	—
**Novices**					
	Task 1	1	45	452,334*.* 29 (209,527*.* 70)	.363
	Task 2	1	45	310,765*.* 11 (198,970*.* 99)	.062
	Task 3	1	45	173,889*.* 87 (73,069*.* 81)	<.001
	Task 4	1	45	161,358*.* 91 (65,405*.* 85)	<.001
	Task 5	1	45	135,273*.* 89 (49,861*.* 64)	<.001
	Task 6	2	44	235,291*.* 93 (167,630*.* 02)	.001
	Task 7	2	44	126,357*.* 86 (59,195*.* 92)	<.001
	Task 8	2	44	188,537*.* 89 (144,107*.* 50)	<.001
	Task 9	2	44	155,625*.* 20 (90,902*.* 41)	<.001
	Task 10	2	44	107,957*.* 18 (54,837*.* 64)	<.001

^a^*P* values compare experts (Task 1) with novices (Tasks 1-10).

**Table 5:** Comparison between the operations of experts at the first task of Session 1 and those of novices at each task.

Sample and task		Session	N	Mean (SD)	*P* value^a^
**Experts**					
	Task 1	1	35	17.49 (11.20)	—
**Novices**					
	Task 1	1	45	17.60 (7.87)	.957
	Task 2	1	45	15.42 (9.39)	.373
	Task 3	1	45	10.84 (3.05)	.002
	Task 4	1	45	12.91 (4.19)	.027
	Task 5	1	45	11.24 (3.68)	.003
	Task 6	2	44	13.89 (6.88)	.101
	Task 7	2	44	11.64 (5.33)	.007
	Task 8	2	44	11.41 (3.87)	.004
	Task 9	2	44	12.46 (5.92)	.020
	Task 10	2	44	11.55 (4.86)	.005

^a^*P* values compare experts (Task 1) with novices (Tasks 1-10).

**Table 6:** Comparisons between the errors of experts for the first task of Session 1 and those of novices at each task.

Sample and task		Session	N	Mean (SD)	*P* value^a^
**Experts**					
	Task 1	1	34	0.35 (0.88)	—
**Novices**					
	Task 1	1	45	1.24 (2.15)	.015
	Task 2	1	45	1.40 (2.33)	.008
	Task 3	1	45	0.80 (1.56)	.112
	Task 4	1	45	1.53 (2.07)	.001
	Task 5	1	45	1.00 (1.83)	.042
	Task 6	2	44	0.86 (1.44)	.058
	Task 7	2	44	0.68 (1.14)	.168
	Task 8	2	44	0.75 (1.28)	.109
	Task 9	2	44	0.84 (1.52)	.101
	Task 10	2	44	0.64 (1.01)	.192

^a^*P* values compare experts (Task 1) with novices (Tasks 1-10).

#### Errors

Table 6 summarizes the comparisons between the errors of experts at the first task of Session 1 and those of novices at each task. Novices made significantly more errors at almost each task of Session 1 (except for Task 3) than did experts at Task 1 of Session 1.

The errors of novices at each task of Session 2 were, however, not significantly different from those of experts at Task 1 of Session 1 (*P*=.015 comparing Task 1 of novices with Task 1 of experts; *P*=.008 comparing Task 2 of novices with Task 1 of experts; *P*=.112 comparing Task 3 of novices with Task 1 of experts; *P*=.001 comparing Task 4 of novices with Task 1 of experts; *P*=.042 comparing Task 5 of novices with Task 1 of experts; *P*=.058 comparing Task 6 of novices with Task 1 of experts; *P*=.168 comparing Task 7 of novices with Task 1 of experts; *P*=.109 comparing Task 8 of novices with Task 1 of experts; *P*=.101 comparing Task 9 of novices with Task 1 of experts; *P*=.192 comparing Task 10 of novices with Task 1 of experts).

## Discussion

This study evaluated the QuestionSys configurator, which was developed to empower end users to develop mobile data collection instruments. In total, 80 participants with and without knowledge of process modeling (ie, experts and novices, respectively) took part and modeled 10 data collection instruments at 2 sessions. Within each session (RQ1 and RQ3), a learning effect was observed: time and number of operations needed to model the data collection instruments became less in each session for novices as well as for experts. Also, across both sessions (RQ4), novices as well as experts improved their needed time and operations, adding further evidence to the mentioned learning effect. Across both sessions, novices also showed a decrease in errors from the first to the last (tenth) data collection instrument. This learning effect across sessions was not observed for experts, probably because they already had very few errors in the first data collection instrument. Yet, errors were not reduced within sessions, indicating that the learning effect regarding reducing errors took more time in novices than the learning effect regarding time and operations.

One week after Session 1 (without using the configurator component), the performances of experts did not change, but needed operations and time increased again in novices (RQ2). This might indicate that the *within-session* learning effect of novices was not as robust to an interval without using the configurator as the within-session learning effect of experts. One reason to explain this result might be that experts work with this type of app on a “day-to-day” basis, retaining some level of expertise between sessions. When novices did not use the configurator for 1 week, they needed to get themselves acquainted with the configurator again, and our results showed that novices got better or faster relatively quickly when they started using the app again. The *decay of learning* noticed within the novices’ sample is a salient factor to be considered and may have practical implications, especially in scenarios requiring infrequent adaptations of instruments. However, as mentioned in the introduction, the QuestionSys configurator addresses the above scenarios in which frequent adaptations of instruments are required. Besides, even for scenarios where infrequent adaptations become necessary, the QuestionSys configurator provides an applicable approach when used by end users experienced in process modeling (ie, experts) as they did not show a decay of learning in this study. However, note that although scenarios with infrequent changes may be supported, they do not constitute the main target of the QuestionSys configurator.

Finally, RQ5 evaluated how many tasks needed to be completed by novices in order to be as good as experts at the first task. Interestingly, novices became significantly faster from the third task on. Moreover, from the third task on, novices needed significantly less operations than experts at the first task, except for Task 6. This might be attributed to the already-mentioned within-session learning effect causing novices to forget how to properly work with the configurator more quickly compared with experts.

Despite the fact that novices did not need to model many data collection instruments in order to be faster and that they needed less operations than experts at the first modeling task, novices were unable to catch up with experts regarding errors. In order to allow for more error-free data collection instruments, more training (than modeling 10 data collection instruments) might be necessary for novices. Furthermore, one could argue that experts are the sample of choice when the data collection instrument should have as less errors as possible.

Several limitations to this study [34] need to be discussed. First, the process of selecting the participants limits external validity or generalizability as mostly students and research associates were recruited for this study. In this context, however, one approach discusses that students may act as proper substitutes in empirical studies [32]. Furthermore, the classification of recruited participants into *novices* and *experts* solely based on a “yes or no” question may be oversimplified and subject for discussion. A more in-depth prequestioning of participants (eg, asking about familiarity with notations such as BPMN, asking for examples of the process models they have created) might allow analysis of how performance depends on the whole spectrum of rated expertise. However, in this study, we aimed to classify novices and experts by distinguishing between no process modeling experiences at all or being in touch with process modeling. Finally, a more elaborated classification by directly observing individuals during modeling or by inspecting the images recorded during the modeling process may be applied in future research. Next, threats to the internal validity constitute the baseline differences between novices and experts regarding gender, education, and field of study. As stated earlier, these differences were intentional as we recruited from different disciplines to be able to compare how well novices could learn using the configurator compared with experts. The target group (end users from medical, psychological, or social sciences) will most likely have no experience in process modeling. Tests measuring processing speed indicated equal cognitive abilities between both groups. Differences in cognitive ability at baseline would be a confounder as this could result in better or faster learnability of the configurator. As another shortcoming, the experts’ sample consisted of less participants than the novices’ sample so that the statistical power was higher in tests for the novices’ sample. Another limitation of this study was that the tasks to be modeled were from various domains (see Table 2). However, the modeling concept used within the configurator is domain agnostic. In order to show the feasibility of this approach for different domains, a vast number of scenarios were modeled. Likewise, the tasks to be modeled did not include modeling of sensors that may be connected to smart mobile devices (eg, to measure vital health care parameters during an interview). In order to deal with these limitations, however, a study specifically targeting health care instruments and the integration of related sensors may be subject for future research.

Despite these limitations, the strength of the study was that we specifically focused on the learnability of the QuestionSys configurator. Note that learnability is a contributory factor to the overall usability. For example, many usability attitude scales explicitly include learnability factors. Interestingly, usability is often measured by subjective reports (eg, usability scales). In this context, studies that measure learnability not by self-reports but by performance measures are more complex and time consuming than those using opinion-based instruments (eg, *System Usability Scale* [SUS]) [35]. Therefore, measuring learnability by performance measures is often neglected in usability studies [36], although it may have an impact on the success or failure of an app [37]. Despite the fact that there exist a plethora of best practices on how to create a user-friendly app, most of them deal with the proper design of the *user interface* [38-40] or with the enhancement of the overall *user experience* [41,42]. Note that there also exist instruments that assess these properties fairly easily (eg, SUS [35]). Although such measures are useful and have proven their applicability in various ways, they might be misleading when evaluating sophisticated apps used by end users with little (or no) IT knowledge. In such scenarios, focusing on the *experience gained* (ie, learning) when continuously working with an app that needs to be evaluated may be more conclusive. However, learning is a process that takes place over time and takes practice into account as well. It can be measured by evaluating the time and effort needed to become better at doing something [43]. Thereby, learnability can be measured using various performance metrics. However, efficiency-based metrics (eg, the *time needed*, *errors committed*, or *operations required*) during task completion are the most common ones.

In summary, the results of this study valuably replicate and extend the results of a previous pilot study [29]. The main findings show that even novices can properly use the configurator and that novices as well as experts perform better when they use the configurator more often (learning effect). Addressing the abovementioned reasons for the lack of sophisticated mobile data collection instruments in large-scale scenarios (see Introduction), the developed configurator component helps build awareness regarding the capabilities of the smart mobile devices used nowadays (see Reason 1). Furthermore, it may allow using sensors during the procedure of collecting data, which may support more complex data collection scenarios (see Reason 2). Most importantly, however, the configurator component not only allows researchers to create data collection instruments themselves but also provides a common (graphical) notation that may improve the communication between researchers and mobile app developers (see Reason 3). Altogether, QuestionSys will significantly influence the way data are collected in large-scale studies (eg, clinical trials). To the best of our knowledge, usability issues in the context of creating mobile data collection apps by researchers have not been studied at this scale previously. Furthermore, this may serve as a valuable benchmark for collecting data in general.

## Appendix

Multimedia Appendix 1 The tutorial screencast for the configurator.

Multimedia Appendix 2 Raw data collected using the configurator.

Multimedia Appendix 3 Description of one task to be modeled (translated from German to English).

## Abbreviations

BPMN: Business Process Modeling and Notation
IT: information technology
RQ: research question
SUS: System Usability Scale
