# Supplementary material for: Learnability of a Configurator Empowering End Users to Create Mobile Data Collection Instruments: Usability Study
Source: JMIR Mhealth Uhealth. 2018 Jun 29;6(6):e148. doi: 10.2196/mhealth.9826 (PMC6045789; doi:10.2196/mhealth.9826)
Supplement: Multimedia Appendix 3 [file mhealth_v6i6e148_app3.pdf]

Task: Patient Information Questionnaire

In this task you should model a questionnaire asking for information needed in the context of a medical intervention (e.g., a gastroscopy) and educate the patient about possible risks.

Carefully read the text before starting modeling. If you have read the task to be modeled, please start the **QuestionSys configurator** application via the provided shortcut on your desktop.

Select the following workspace:

Workspace: **Study**

Questionnaire: **Patient Information**

Open the "**Editor**" view and start modeling the respective questionnaire. Save the final model to the desktop of your computer.

1. The first page of the questionnaire contains a headline and text element with general information regarding the upcoming medical intervention (e.g., gastroscopy). Furthermore, demographic information of the patient (e.g., name, age, gender, …) shall be collected. Additionally, the patient should answer, whether a family member shall be contacted after the intervention. If "yes", the patient shall continue with page 2, otherwise with page 3.
2. The second page shall only be displayed if the patient wants a family member to be informed regarding the course of the intervention. This page shall ask about details of the person to be informed (e.g., name, phone number, …).
3. Regardless of whether one should be informed, the 3rd page will be displayed next. This page contains a text if the patient wishes to be anesthetized or not. Thereby, the following options may be available: none, local, full.
4. This page, in turn, shall only be displayed if the intervention takes place under local anesthesia. Thereby, an additional form shall be displayed to provide information regarding possible risks. The patient has to sign this form in order to continue.
5. This page, in turn, shall only be displayed if the intervention takes place under full anesthesia. It shall display similar information as described before, however, texts shall be adapted in order to reflect the given circumstances.

Mental Effort: Patient Information Questionnaire

Answer the following questions:

1. The mental effort for creating the model was considerably high.

strongly agree	agree	rather agree	neutral	rather disagree	disagree	strongly disagree
o	o	o	o	o	o	o

2. I was able to properly solve the given task.

strongly agree	agree	rather agree	neutral	rather disagree	disagree	strongly disagree
o	o	o	o	o	o	o

3. The task was rather difficult.

strongly agree	agree	rather agree	neutral	rather disagree	disagree	strongly disagree
o	o	o	o	o	o	o

4. I had to concentrate myself when creating the model.

strongly agree	agree	rather agree	neutral	rather disagree	disagree	strongly disagree
o	o	o	o	o	o	o

5. Creating the model was exhausting.

strongly agree	agree	rather agree	neutral	rather disagree	disagree	strongly disagree
o	o	o	o	o	o	o
